# Comparative analysis of programmed cell death pathways in filamentous fungi

**DOI:** 10.1186/1471-2164-6-177

**Published:** 2005-12-08

**Authors:** Natalie D Fedorova, Jonathan H Badger, Geoff D Robson, Jennifer R Wortman, William C Nierman

**Affiliations:** 1 The Institute for Genomic Research, 9712 Medical Center Drive, Rockville, MD 20850, USA; 2 Faculty of Life Sciences, 1.800 Stopford Building, University of Manchester, Manchester M13 9PT, UK; 3 The George Washington University School of Medicine, Department of Biochemistry and Molecular Biology, 2300 Eye Street, NW Washington, DC 20837, USA

## Abstract

**Background:**

Fungi can undergo autophagic- or apoptotic-type programmed cell death (PCD) on exposure to antifungal agents, developmental signals, and stress factors. Filamentous fungi can also exhibit a form of cell death called heterokaryon incompatibility (HI) triggered by fusion between two genetically incompatible individuals. With the availability of recently sequenced genomes of *Aspergillus fumigatus *and several related species, we were able to define putative components of fungi-specific death pathways and the ancestral core apoptotic machinery shared by all fungi and metazoa.

**Results:**

Phylogenetic profiling of HI-associated proteins from four Aspergilli and seven other fungal species revealed lineage-specific protein families, orphan genes, and core genes conserved across all fungi and metazoa. The Aspergilli-specific domain architectures include NACHT family NTPases, which may function as key integrators of stress and nutrient availability signals. They are often found fused to putative effector domains such as Pfs, SesB/LipA, and a newly identified domain, HET-s/LopB. Many putative HI inducers and mediators are specific to filamentous fungi and not found in unicellular yeasts. In addition to their role in HI, several of them appear to be involved in regulation of cell cycle, development and sexual differentiation. Finally, the Aspergilli possess many putative downstream components of the mammalian apoptotic machinery including several proteins not found in the model yeast, *Saccharomyces cerevisiae*.

**Conclusion:**

Our analysis identified more than 100 putative PCD associated genes in the Aspergilli, which may help expand the range of currently available treatments for aspergillosis and other invasive fungal diseases. The list includes species-specific protein families as well as conserved core components of the ancestral PCD machinery shared by fungi and metazoa.

## Background

*Aspergillus fumigatus *is the most prevalent causative agent of invasive aspergillosis in immunocompromised patients and it can also cause asthma, allergies, and mycotoxicosis [1]. Other species of this genus including *Neosartorya fischeri *(teleomorph of *A. fischerianus*), *A. flavus*, *A. terreus*, *A. niger*, and *A. nidulans *can also cause diseases in humans, animals and plants all over the world [2]. Despite the medical and agricultural importance of this genus, limited antifungal therapies are available due to high toxicity, low efficacy rates, and growing drug resistance [3]. Activation of endogenous programmed cell death (PCD) reactions is a promising approach to combat invasive aspergillosis and other fungal diseases. Several antifungal agents including amphotericin B and rapamycin have been shown to induce cell death cascades in filamentous fungi [4-7]. Better understanding of these pathways might provide the basis for the development of novel anti-fungal therapeutics against aspergillosis and give further insights into evolution of programmed cell death in the eukaryotic cell.

Several different cell death programs seem to exist in parallel in fungi and often resemble mammalian apoptosis and yeast autophagy [8-11]. The best studied form of programmed cell death in filamentous fungi is heterokaryon incompatibility (HI) that can be triggered by cellular fusions between hyphae of incompatible individuals during vegetative growth or between incompatible germlings during the establishment of fungal colonies [12]. These fusion between two individuals with incompatible *het *(heterokaryon incompatibility) loci triggers the HI reaction characterized by growth inhibition, repression of asexual sporulation, hyphal compartmentation and death in the heterokaryotic cell [13,14]. Although HI is ubiquitous in filamentous fungi, its biological significance and evolutionary origin is still unknown. It has been proposed to serve as a self/nonself recognition system responsible for limiting genetic exchange and horizontal transfer of cytoplasmic infectious elements [15-17]. Coincidentally, one of the HI inducers in *P. anserina *is a prion capable of infectious propagation [18].

Much of what is known about the underlying mechanisms of programmed cell death in fungi comes from only three species, *Saccharomyces cerevisiae*, *P. anserina *and *N. crassa*. With the availability of the newly sequenced genomes of *A. nidulans *(J. Galagan et al., in press), *A. oryzae *(M. Machida et al., in press), *A. fumigatus *Af293 (W. Nierman et al., in press), and *N. fischeri *(W. Nierman, unpublished) we were able to identify their putative PCD effectors and mediators. In this study, we applied a BLASTp-plus-phylogeny reconstruction approach to survey the *Aspergillus spp*. genomes for homologs of characterized programmed cell death proteins from fungi and animals. Several fungi-specific families as well as components of the core cell death machinery shared by filamentous fungi and metazoan were identified.

## Results and discussion

### Inducers of HI-triggered PCD

Several components of the HI reaction encoded by the *het *genes, the HI suppressor genes, and the HI-induced genes have been characterized at the molecular level in *P. anserin*a and *N. crassa*. In natural populations of *P. anserina*, *N. crassa *and *Cryphonectria parasitica*, the functional *het *genes exist as two or more polymorphic alleles conferring alternative specificities [19-22]. Different loci may be responsible for triggering the HI reaction in these two and other fungal species. Heterocomplex formation between alternative *het *gene products is thought be a common theme in nonself recognition during allelic and non-allelic HI [23,24]. One of the genetically (and potentially physically) interacting partners is often a HET domain protein [15] that may interact with well-conserved proteins playing important roles in development and differentiation (Dementhon and L. Glass, unpublished).

Although at least eight loci have been implicated in HI in *A. nidulans *[25], none of them has been characterized at the molecular level. To identify putative HI-associated proteins in the Aspergilli, we first searched completely sequenced fungal genomes using known inducers and mediators of heterokaryon incompatibility as BLASTp queries. We examined the domain composition and phyletic distribution of the BLASTp hits and built phylogenetic trees for several protein families. We also applied domain fusion analysis to several so called Rosetta Stone proteins with unusual domain composition to infer protein domain interactions and functional linkage between putative HI-associated proteins identified in the Aspergilli proteomes.

### HET-C2 proteins

**Figure 1:**
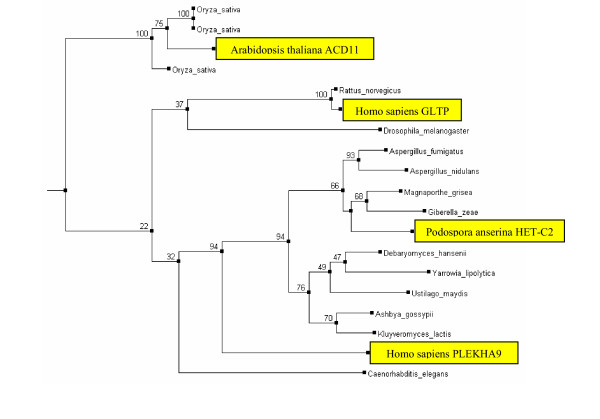
**Phylogenetic tree of the HET-C2/GLTP/ACD11 family of proteins. **Tree reconstruction was performed as described in the Methods section. Experimentally characterized proteins are shown in yellow. The numbers indicate percent bootstrap values for selected internal branches. Hemiascomycete members including *Debaryomyces hansenii, Ashbya gossypii, Yarrowia lipolytica, and Kluyveromyces lactis *clustered together with the basidiomycete *U. maydis*.

Our database BLASTp searches identified orthologs of *P. anserina *HET-C2 [26], in all filamentous fungal genomes sequenced to date (Table 1). The family also has a high level of sequence conservation and wide phyletic distribution in other taxa. HET-C2 orthologs are found in several Saccharomycetes, including *Debaryomyces hansenii *and *Kluyveromyces lactis*, (Fig. 1). Yet no homologs are detected in *S. cerevisiae *and *S. pombe*, suggesting a gene loss in some yeast lineages. HET-C2 homologs are also present in most animals and plants.

**Table 1:** Putative HI inducers

**Protein/Domain**	**ASP**	**SOR**	**Scer**	**Spom**	**BAS**	**Biological function/process**
HET-C	1	1	0	0	1	Unknown
RNR1	1	1	1	1	1	Cell cycle control
HET-C2	1	1	0	0	1	Cell cycle control^a^, sphingolipid sensing
MAT-1/2	1–2	1	2	2	0	Regulation of sexual differentiation
HET	7–38	38–94	0	0	0	Signaling, regulation of sexual differentiation
HET-s/LopB	0–1	1–7	0	0	0	Unknown
NACHT	12–19	2–18	0	0	0	Signaling, NTP binding, oligomerization
Pfs+NACHT	5–7	1	0	0	0	Signaling, nucleoside metabolism

ASP, *A. fumigatus*, *N. fischeri*, *A. oryzae*, *A. nidulans*; SOR, *N. crassa*, *G. zeae*, and *M. grisea*; BAS: *Cryptococcus neoformans *and *Ustilago maydis*; Scer, *S. cerevisiae*; Spom, *Schizosaccharomyces pombe*; ^a^, putative function.

The high level of conservation among the HET-C2 family members is consistent with the important role these proteins may play in the glycosphingolipid and sphingosine metabolism and possibly in regulation of cellular stress responses. HET-C2 shows significant similarity to human GLTP [27] and *Arabidopsis thaliana *ACD11 [28] proteins, which catalyze the intermembrane transfer of glycosphingolipids and sphingosines, respectively. ACD11 has also been shown to function in PCD and pathogen defense in plants. In *Aspergilli*, sphingosines have been also shown to induce an apoptosis-like PCD [4] and to affect cell cycle progression [29]. *P. anserina *HET-C2 was proposed to act as a glycolipid metabolite sensor in addition to its role in glycolipid transfer, regulation of ascospore maturation, and triggering HI [26,30]. The high level of sequence conservation in this family, suggests that the role of HET-C2 orthologs in the Aspergilli PCD pathway is likely to be similar.

### HET-C proteins

**Figure 2:**
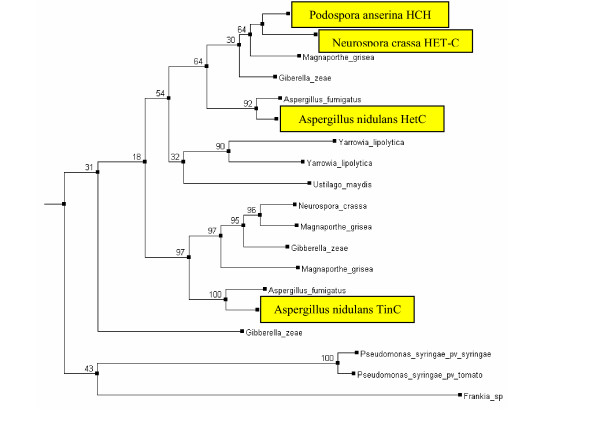
**Phylogenetic tree of the HET-C family of proteins. **Tree reconstruction was performed as described in the Methods section. Experimentally characterized proteins are shown in yellow. The numbers indicate percent bootstrap values for selected internal branches.

Further analysis showed that all *Aspergillus *species have direct orthologs of *N. crassa *HET-C [31] and its close homolog of unknown function (Table 1). The phylogenetic tree has a bipartite division resulting from an early gene duplication event predating the separation of Eurotiomycetes and Sordariomycetes (Fig. 2). HET-C is orthologous to *A. nidulans *HetC and homologous to TinC [32]. The HET-C domain is found in many ascomycetes and basidiomycete species, but surprisingly in only one yeast species, *Yarrowia lipolytica*, which clusters together with HET-C homologs from basidiomycete species. Unexpectedly, a partial HET-C domain is also present in several epiphytic and symbiotic bacteria including two gamma-proteobacteria, *Pseudomonas syringae *[GenBank: AAY39263 and GenBank: AAO58004], a cyanobacterium, *Nostoc punctiforme *[GenBank: ZP_00106220], and an actinomycete of the genus Frankia [GenBank: ZP_00567177]. The phylogenetic tree of the conserved N-terminal portion of HET-C shows that the bacterial proteins form a coherent clade with a long branch connecting it to the rest of the tree. We had to exclude the *Nostoc punctiforme *protein from the phylogenetic analysis because it was too divergent, but it also showed more similarity to the bacterial proteins.

Based on the current tree topology, the origin of the bacterial homologs is not clear. It can be attributed to vertical inheritance from the last common ancestor between bacteria and fungi followed by massive gene loss in most bacterial and yeast lineages. Alternatively, it can be explained by horizontal transfer of the ancestral *het-C *gene from epiphytic fungi followed by rapid divergence in bacteria. In the latter case, the gene must have persisted in bacterial populations by conferring a selective advantage to the recipients. Since heterologous expression of an *N. crassa het-C *allele was also shown to trigger an HI-like growth defect in *P. anserina *[21], the *het-C *homologs in *P. syringae *or related species may induce growth inhibition in epiphytic filamentous fungi and thus facilitate substrate defense.

*N. crassa *polypeptides encoded by the *het-C *alleles of alternative specificity were shown to form a heterocomplex localized to the plasma membrane during the HI reaction [23]. It has a putative signal peptide, a conserved HET-C domain and a divergent C-terminal glycine-rich region, often found in extracellular glycoproteins. The biological role of the HetC proteins in the Aspergilli is unknown. *A. nidulans *TinC has been shown to stabilize the NimA mitotic kinase required for mitotic entry [32]. *A. nidulans *strains lacking *tinC *displayed cold and osmotic sensitivity and overexpression of its truncated form produced growth inhibition, defects in nuclear envelope fission and cell cycle [32]. It is unlikely that either protein triggers the HI reaction in the *A. nidulans*. Moreover, *het-C *may not act as a *bona fide het *gene in other fungal species, since no *het-C *polymorphism was observed in *A. flavus *(K. Ehrlich and P. Cotty, unpublished),* A. nidulans *[32], and *P. anserina *[21] isolates. Nonetheless, expression of the *N. crassa *het-c(PA) allele triggers a growth inhibition similar to the HI reaction in *P. anserina*. If *N. crassa *HET-C has a similar role to TinC, it may explain the growth inhibition effects caused by expression of *N. crassa *(and possibly bacterial) *het-c *in *P. anserina *[21].

### HET domain proteins

HET domain [15,33] proteins were identified using the HMMer package as described in Methods. Unlike the ubiquitous HET-C2 family, the HET domain appears to be limited to filamentous ascomycetes and is not detected in yeasts or basidiomycete species (Table 1). In the Aspergilli, the number of HET domain proteins varies from seven in *N. fischeri *to 38 in *A. oryzae*. The tree topology delineates multiple duplication events in filamentous ascomycetes species followed by rapid diversification and gene loss in several *Aspergillus spp*. (data not shown). Orthologous relationships within this Aspergillus family are difficult to establish, except for a subfamily of HET and Ankyrin domain proteins, which appear to be related by direct vertical descent (data not shown).

**Figure 3:**
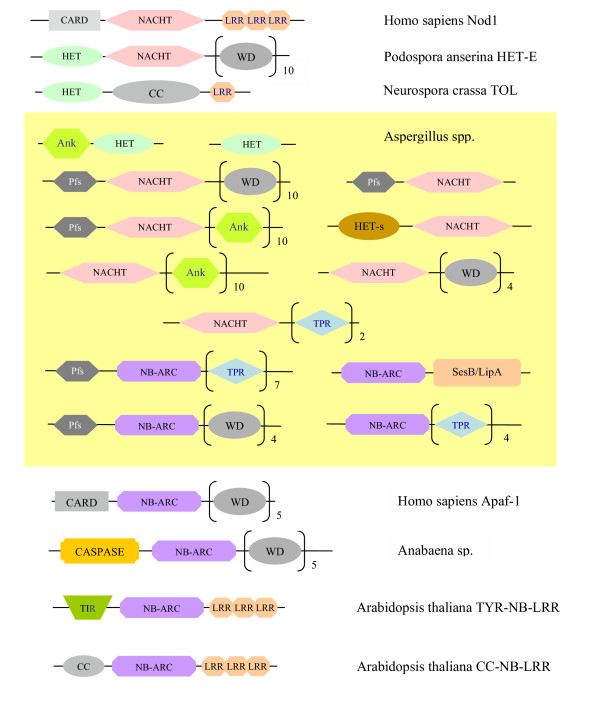
**Domain organization of NACHT, HET-s/LopB, and HET domain proteins. **Each shape indicates a specific conserved domain. Fused domains that form a single polypeptide chain are connected by a horizontal line. Aspergilli proteins are located in the area with the yellow background. Ank, Ankyrin domain; CARD, caspase recruitment domain; CC, coiled coil domain; HET, HET domain; HET-s/LopB, new domain found in HET-s and LopB proteins; LRR, leucine-rich repeat; NACHT, NACHT domain; NB-ARC, NB-ARC domain; Pfs, nucleoside phosphorylase domain; SesB/LipA, SesB/LipA domain, found in putative serine esterases and in signaling protein SesB from *Nectria haematococca*; TIR, toll-interleukin receptor domain, TPR, tetratricopeptide repeat; WD, WD40 domain, found in eukaryotic proteins with various functions including adaptor/regulatory modules in signal transduction; typically contains the WD dipeptide at its C-terminus and is 40 residues long. Figure is not drawn to scale.

The HET domain expansion in filamentous ascomycetes may represent a niche adaptation strategy to process a large number of similar stimuli associated with defense against pathogens, self/nonself recognition, differentiation, or analogous roles. It is found in *N. crassa *HET-6 and TOL and in *P. anserina *HET-D and HET-E, and so appears to be critical to the HI reaction in both species (for review see [15]). In *P. anserina *HET-D and HET-E, HET domains are followed by a NACHT domain and multiple WD repeats, while *N. crassa *proteins contain a coiled-coil domain and LRR repeats, instead (see Figure 3). In addition to HET-6 and TOL, *N. crassa *has about 50 other HET domain proteins, whose role in the HI reaction if any is as yet unknown.

### Identification of the HET-s/LopB domain

**Figure 4:**
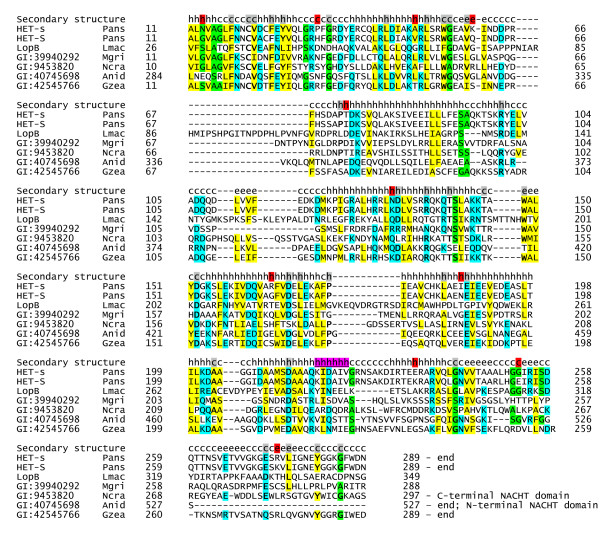
**Multiple alignment of the HET-s/LopB protein family. **The first line in the alignment shows the prediction of secondary structure content: h for helical, e for extended, c for coiled. Residues conserved among several proteins are marked with gray shading. The polymorphic positions in HET-s and HET-S proteins are shown with red shading. Proteins are listed under their unique GenBank identifiers (first left column). Species are indicated in the second from left column: Pans, *Podospora anserina*; Lmac, *Leptosphaeria maculans*; Mgri, *Magnaporthe grisea*; Ncra, *Neurospora crassa*; Anid, *Aspergillus nidulans*, Gzea, *Gibberella zeae*. Yellow shading indicates uncharged amino acids (A, I, L, V, M, F, Y, or W). Conserved small residues (G, A, or S) are shown in green. Charged residues (D, E, K, R, N, or Q) are shown in blue. The residues corresponding to the proteinase K-resistant amyloid core in *P. anserina *Het-s are highlighted in purple and underscored.

Initial BLASTp searches using the *P. anserina *HET-s sequence [34] as a query revealed homologs in *A. nidulans, P. chrysogenum, M. grisea*, *N. crassa *and *G. zeae *(Table 1). Iterative PSI-BLAST searches identified a new domain that includes more proteins from the same species plus a pathogenicity protein, LopB, from the Dothideomycete fungus *Leptosphaeria maculans *[35]. For LopB and most other members of this family, sequence similarity is limited to the N-terminal globular domain of HET-s (Fig. 4) [36]. Two members from *A. nidulans *and *N. crassa *have an adjacent NACHT domain (described below) at the N- and C-terminus, respectively.

As mentioned earlier, HI was proposed to act as a self/nonself recognition system responsible for limiting the spread of numerous infectious elements in natural populations [15-17]. Coincidentally, HET-s prion behaves as a non-conventional infectious element capable of propagation during anastomosis and sexual reproduction in *P. anserina *[37]. HET-s can exist in two forms: as a normal protein [18] and as an infectious prion [38], capable of propagating as a self-perpetuating amyloid aggregate [18,36]. Its rather unexpected similarity to LopB implies that members of the family may have another function unrelated to HI. Although its specific role in *L. maculans *is unknown, *LopB*^- ^mutants showed impaired ability to form lesions on oilseed rape [35]. LopB contains a predicted signal peptide suggesting that it is secreted and might contribute to the *L. maculans *pathogenicity by compromising host membranes. The fusions between HET-s/LopB and NACHT domain in *N. crassa *and *A. nidulans *suggests that, in other species, proteins containing one of these two domains may physically interact.

### STAND domain proteins

Using *P. anserina *HET-E as a BLASTp query, we identified several proteins containing NACHT domain in the Aspergilli and other filamentous ascomycetes. Further HMMer searches detected two Aspergillus-specific expansions of the STAND domain [39]: NACHT NTPases and NB-ARC ATPases (Table 1). These NTP-binding proteins are often linked to various protein-binding modules such as WD40, Ankyrin or TPR at the C-terminus and a highly divergent nucleoside phosphorylase (Pfs) domain at their N-terminus (Fig. 3). A different type of domain composition is found in several other STAND NTPases. NB-ARC can fused to a LipA domain found in putative serine esterases and in the SesB protein from *Nectria haematococca *[40]. Some NACHT NTPases are linked to the HET-s/LopB domain described above. The orthologous relationships within the two STAND domain expansions are difficult to establish since the proteins are highly divergent and exhibit uneven phyletic distribution. They also appear to have undergone multiple domain shuffling events as well as lineage-specific gene loss and expansions during the evolution of the Aspergilli as well as other filamentous fungi.

As mentioned earlier, one of *N. crassa *NACHT domain protein is linked to the HET-s/LopB domain. *P. anserina *HET-D and HET-E are fused to the HET domain and 11 WD40 repeats, which determine their allelic specificity [41]. HET-E has been shown to genetically (and potentially physically) interact with HET-C2 to trigger incompatibility in *P. anserina *suggesting that the interaction may activate the ceramide stress response pathway [24]. Similar to the HET domain expansion, the two STAND domain expansions may represent a niche adaptation strategy in filamentous ascomycetes. The multiple fusions involving STAND domains in filamentous fungi may be responsible for the enhancement of their repertoire of signal-transducing interactions, linking preexisting signaling pathways, or integrating multiple signals.

Although their specific biological role is unknown, several functional inferences can be made regarding the role of the STAND domain proteins in the Aspergilli. Despite the variability of their domain architecture, the regulatory/signaling function of STAND NTPases seems to be conserved from fungi to man to possibly bacteria. The domain has been implicated in hetero-oligomerization and signal transduction during apoptosis, inflammatory and pathogen responses in animals and plants, and in transcriptional regulation of secondary metabolism in bacteria [42-44]. Similar to their fungal counterparts, animal and plant members of the superfamily tend to be fused to death effector domains at the N-terminus and to repetitive protein binding/regulatory modules at the C-terminus [39].

Other observations suggest that Pfs and STAND domain fusion proteins in the Aspergilli may play a regulatory or signaling role. In plants, the Pfs domain was found in several stress-inducible enzymes [42]. The Pfs domain is also found in bacterial methylthioadenosine/S-adenosylhomocysteine (MTA/SAH) nucleosidases and phosphoribosyltransferases [45]. The bacterial nucleosidases function in methionine salvage pathway and control intracellular levels of MTA/SAH and, in several cases, production of a quorum-sensing signaling molecule [46]. In animals, MTA has been shown to affect many critical responses including regulation of gene expression, proliferation, differentiation and apoptosis [47].

The NB-ARC and LipA as well as NACHT and HET-s/LopB domain fusion proteins may function as bistable switches in signaling cascades. A putative serine esterase domain, LipA, is found in the SesB protein implicated in bistability of developmental signaling cascades in *Nectria haematococca *[40]. Incidentally, *sesB *is adjacent to a gene, which encodes a NACHT and Ankyrin domain protein. HET-s is also associated with bistability of the HI reaction resulting from the spread of the infectious prion in *P. anserina *[38].

Another line of evidence implicating this family in integration of developmental, stress, and nutrient availability signals, comes from expression data. At least five NACHT domain proteins appear to be regulated by LaeA in *A. fumigatus *(N. Keller, S. Kim and W. Nierman, unpublished), a putative chromatin-dependent regulator of secondary metabolism, virulence and conidiation [48,49]. A few other putative PCD-associated proteins seem to be affected by LaeA in *A. fumigatus*, including both metacaspases, bZIP transcription factor JlbA, BAX Inhibitor family protein, Cu^2+^/Zn^2+ ^superoxide dismutase SOD1, histone chaperone ASF1, and AMID-like mitochondrial oxidoreductase, some of which are described below.

### Mediators of HI-triggered PCD

In addition to the putative HI inducers, the Aspergilli possess homologs of HI suppressors from *P. anserina *and *N. crassa *(Table 2) [15]. Thus, sequence similarity searches detected orthologs of *N. crassa *VIB-1 [50] and *P. anserina *MOD-A, MOD-D and MOD-E [51-53]. VIB-1, a putative regulator of conidiation and HI in *N. crassa*, is orthologous to *Penicillium chrysogenum *PhoG and *A. nidulans *PacG, which were annotated as putative non-repressible acid phosphatases via transformation experiments [50,54,55]. There is no apparent orthologs in yeasts, although, VIB-1 is a distant homolog of the Ndt80p transcription factor from *S. cerevisiae *[56]. The distribution of MOD-A orthologs also appears to be limited to filamentous ascomycetes. Only *A. oryzae*, but not other Aspergilli, contains an ortholog of MOD-A, implicated in ascomycete-specific functions such as regulation of growth arrest during HI and female organ formation in *P. anserina *[51]. On the contrary, MOD-D and MOD-E display a very high level of sequence conservation and have a much wider phyletic distribution with homologs in all fungi and higher eukaryotes. MOD-D, a G protein alpha subunit, is orthologous to GpaB and GanB from *A. fumigatus *and *A. nidulans*, respectively [57,58].

**Table 2:** Putative HI mediators

**Protein**	**ASP**	**SOR**	**Scer**	**Spom**	**BAS**	**Biological function**
IDI-1	0	0	0	0	0	Unknown
IDI-2	0–1	0–1	0	0	0	Unknown
IDI-3	0	0	0	0	0	Unknown
IDI-4, JlbA	1	1	0	0	0	Regulation of transcription in response to nutritional stress
VIB-1	1–2	1	0	0	0	Regulation of sporulation
MOD-D, GpaB, GanB	1	1	1	0	1	Regulation of asexual sporulation, pathogenicity, G-protein signaling
MOD-A	0–1	1–3	0	0	0	Regulation of sexual differentiation
MOD-E, HSP90	1	1	2	1	2	Regulation of sexual differentiation, life span, protein folding
IDI-7, AUT7	1	1	1	1	1	Regulation of sexual differentiation, autophagy
IDI-6, Alp2p	1	1	2	2	1	Regulation of sexual differentiation, autophagy

ASP, *A. fumigatus*, *N. fischeri*, *A. oryzae*, *A. nidulans*; SOR, *N. crassa*, *G. zeae*, and *M. grisea*; BAS: *C. neoformans *and *U. maydis*; Scer, *S. cerevisiae*; Spom, *S. pombe*.

The degree of sequence conservation seems to be linked to the relative importance of the biological function and more conserved proteins appear to be functional orthologs. The similarity between VIB-1 and Ndt80p, a transcription factor involved in regulation of sporulation and meiosis in *S. cerevisiae *[56], suggests that *phoG *and *pacG *may encode a transcriptional regulators of the acid phosphatase, rather than the enzyme itself [59]. MOD-D and MOD-E have been shown to function as an alpha subunit of heterotrimeric G protein, and an HSP90 family molecular chaperone, respectively [52,53]. *P. anserina *MOD-E, in addition to suppressing HI, is involved in regulation of development and the sexual cycle [53,60]. The MOD-E/HSP90 function during the sexual cycle appears to be conserved from fungi to mammals [61]. Mammalian HSP90 family chaperones also mediate the unfolded protein response to endoplasmic reticulum stress through regulation of the secretory pathway, cell cycle and programmed cell death [61,62].

Besides HI suppressors, *Aspergilli *also possess orthologs of *P. anserina idi*-2, *idi*-4, *idi*-6 and *idi*-7 genes (Table 2) induced by heterokaryon incompatibility and implicated in autophagy in response to starvation and sporulation and in regulation of sexual differentiation [8,63-65]. IDI-6 and IDI-7 proteins seem to be highly conserved across fungal species; while IDI-2 and IDI-4 are poorly conserved and their distribution is limited to filamentous fungi. No detectable homologs of IDI-1 and IDI-3 are found in Aspergilli and an IDI-2 ortholog is only present in *A. oryzae*. Autophagic serine protease IDI-6 is orthologous to *A. fumigatus *Alp2 that has been shown to function in regulation of sporulation as well as pathogenesis in *A. fumigatus *[66]. Likewise, IDI-4 an ortholog of the *A. fumigatus *JlbA, a putative bZIP transcription factor induced by amino acid starvation [67].

Many HI-associated genes have wide phyletic distribution and are well-conserved across filamentous fungi. Some are involved in autophagy, suggesting that the incompatibility function might have evolved by recruiting components of the cellular system controlling adaptation to starvation [11]. In addition, cytological alterations during HI in *P. anserina *are similar to those observed during starvation and treatment with rapamycin, an inhibitor of the TOR (target of rapamycin) signaling pathway that controls autophagic degradation in *S. cerevisiae *[68]. Yet, many seem to perform unrelated functions such as regulation of development or sexual differentiation, implying that heterokaryon incompatibility may have utilized components of these cell programs as well.

### Downstream PCD machinery

In addition to HI, filamentous fungi appear to possess a wide range of PCD reactions triggered by various death stimuli. In Aspergilli, the apoptotic-like phenotypes and are observed during entry into stationary phase and sporulation and on exposure to certain antifungal agents, peptides, and sphingosines [4,6,7,69,70]. Similarly, in *N. crassa*, morphological changes during the HI reaction, starvation, and DNA damage response resemble apoptosis [9,10,71]. The apoptotic machinery of filamentous fungi may share some key components with yeast and mammalian systems. First, we looked for homologs of apoptotic proteins in *S. cerevisiae*, which can undergo PCD in response to nutritional and oxidative stresses, plant antifungal peptides, hydrogen peroxide, or during aging and mating [72-75].

To identify candidate apoptosis-associated proteins in the Aspergilli, BLASTp similarity searches were performed with yeast apoptotic proteins as queries. Homologs of more than 30 yeast proteins were detected including metacaspases and caspase-regulating serine protease HtrA2 (Table 3). Phylogenetic analysis shows that many Aspergillus proteins are in one-on-one orthologous relationships with *S. cerevisiae*, *S. pombe*, and basidiomycete proteins (data not shown).

**Table 3:** Putative apoptotic mediators (fungal protein homologs)

**Protein**	**ASP**	**SOR**	**Scer**	**Spom**	**BAS**	**Biological function/process**
Aif1p	0	0	1	0	0	Caspase independent apoptosis
Stm1p	1	1	1	1	1	Caspase independent apoptosis
Cdc6p	1	1	1	1	1	Cell cycle control, DNA replication
Asf1p	1	0–1	1	1	1	Cell cycle, chromatin assembly, mating
Cdc13p	0	0	1	0	0	Cell cycle control, telomere-binding
Cyc1p	1	1	1	1	1	Electron transport, cytochrome c
Mre11p	1	1	1	1	1	Maintenance of genome integrity
Rad50p	1	1	1	1	1	Maintenance of genome integrity
Yca1p	2–3	1–4	1	1	1–2	Metacaspase
Sfk1p	1	1	1	1	1	Mitochondrial death pathway
Atp4p	1	1	1	1	1	Mitochondrial F1F0 ATP synthase
DAP3	1	1	1	1	1	Mitochondrial fragmentation
HtrA2	1	1–2	1	2	0	Mitochondrial homeostasis
Lsm4p	1	1	1	1	1	mRNA processing
Nsr1p	1	1	1	1	1	rRNA processing
Hel10p	1	0–1	1	0	0	Apoptosis
Uth1p	1	1	4	2	0	Response to stress
Sod1p	1	1	1	1	1	Response to stress
BI-1	1	1	1	1	1	Response to stress
Mras1	1	1	1	1	1	Regulation of development, signaling
FadA/GpaA	1	1	1	1	1	Regulation of sexual differentiation, sporulation, G-protein signaling
Ste4p/CGB1	1	1	1	1	1	Regulation of sexual differentiation, sporulation, G-protein signaling
Ste18p	1	1	1	1	1	Regulation of sexual differentiation
Ste20p	1	0–1	1	1	1	Regulation of sexual differentiation
Sip3p	1	1	2	1	0	Regulation of sexual differentiation
Sst2p, FlbA	1	1–2	1	1	1	Regulation of sexual differentiation
Oxa1	1	1	1	1	1	Regulation of life span, respiratory complex assembly
RMP1	1	1	1	1	1	Regulation of development and life span, respiratory complex assembly
Lag1p	1	1	1	2	1	Sphingolipid-mediated signaling
Sar1p, SarA	1	1	1	1	1	Ubiquitin-proteosome system
Cdc48p	1	1	1	1	1	Ubiquitin-proteosome system
Ubp10p	0	0–1	1	0	0	Ubiquitin-proteosome system
Ppa1p	1	1	1	1	0	Vacuolar ATPase subunit

ASP, *A. fumigatus*, *N. fischeri*, *A. oryzae*, *A. nidulans*; SOR, *N. crassa*, *G. zeae*, and *M. grisea*; BAS: *C. neoformans *and *U. maydis*; Scer, *S. cerevisiae*; Spom, *S. pombe*.

BLASTp and HMM searches identified homologs of ~50 human and mouse apoptotic proteins in the Aspergilli [see Additional file 1 ]. Similar to yeasts, filamentous ascomycetes lack the upstream metazoan apoptotic regulators including members of the bax/bcl-2 family and p53, while downstream components of the apoptotic machinery appear to be shared. Interestingly, many *Aspergillus *proteins are more similar to their human counterparts such as AMID, BIR1, HtrA, and CulA, than to yeast homologs. Thus, the tree topology of the AIF family confirmed that Aspergilli proteins are more closely related to human AMID than to *S. cerevisiae *Aif1p, which clustered together with plant homologs [see Additional file 2 ]. Moreover, homologs of several key components of the mammalian apoptotic machinery, including AmsH and Poly(ADP-ribose) polymerase (PARP), are not detected in *S. cerevisiae *(Table 4).

**Table 4:** Putative apoptotic mediators absent in *S. cerevisiae*

**Protein/Domain**	**ASP**	**SOR**	**Scer**	**Spom**	**BAS**	**Biological function/process**
TRAF-3	1	1	0	0	1	Caspase dependent apoptosis
Mst3/STK24	1	0–1	0	2	0–1	Caspase dependent apoptosis
PARP	1	1	0	0	0	Caspase independent apoptosis
AMID	1–2	0–1	0	0	1	Caspase independent apoptosis
GRIM-19	1	1	0	0	1–2	Electron transport, NADH ubiquinone oxidoreductase
NDUFS1	1	1	0	0	1	Electron transport, NADH ubiquinone oxidoreductase
APAF	3–8	0–2	0	0	0	NTP binding, hetero-oligomerization
12R-LO	1	0–1	0	0	0	Peroxidation of arachidonic acid
15-LO	1	0–1	0	0	0	Peroxidation of arachidonic acid
PTDSR/PSR	1	1	0	0	1	Recognition of apoptotic cells

ASP, *A. fumigatus*, *N. fischeri*, *A. oryzae*, *A. nidulans*; SOR, *N. crassa*, *G. zeae*, and *M. grisea*; BAS: *C. neoformans *and *U. maydis*; Scer, *S. cerevisiae*; Spom, *S. pombe*.

At least two Aspergilli proteins appear to be functional homologs to their mammalian counterparts. The enhanced PARP and caspase-like activity reported in *A. nidulans *during sporulation-induced PCD is consistent with the presence of both metacaspase-dependent and -independent apoptotic pathways [70,76]. In addition, the metacaspase-independent apoptosis pathway was shown to operate in *A. fumigatus *during stationary phase and treatment with fungicidal sphingoid bases and antifungal agents [4,69]. For the rest of the fungal proteins, further experimental characterization is required before any conclusions can be drawn regarding their involvement in PCD. Many yeast and mammalian apoptotic proteins appear to be involved in regulation of cell programs monitoring the cell status such as maintenance of genome integrity, cell cycle control, glycolipid metabolism, and ubiquitin-dependent proteolysis [see Additional file 2] [77]. It is likely that at least non-apoptotic function is conserved in both fungi and metazoa. The results also support the idea that complex development and differentiation in filamentous fungi may require additional PCD pathways or their components not found in unicellular yeasts [78].

## Conclusion

Our analysis identified more than 100 putative PCD-associated genes in this genus, suggesting a complex uncharacterized regulatory network. Their further characterization may help expand the range of currently available treatments for invasive aspergillosis. The list includes lineage-specific protein families as well as conserved core components of the ancestral PCD machinery shared by all fungi and metazoa.

The most divergent group is comprised of putative HI inducers such as STAND, HET-s/LopB and HET domain proteins that show extreme variability in sequence, copy number and domain composition. The STAND NTPases are predicted to interact with different types of effector/signaling components and function as key integrators of stress and nutrient availability signals. On the other end of the spectrum are HI-associated proteins that show broad phyletic distribution and high sequence conservation. They tend to be involved in regulation of development, sexual differentiation, and stress reactions, suggesting that the HI function in filamentous fungi may have evolved by recruiting components from these preexisting pathways [11,52,53].

Further analysis revealed homologs of the yeast PCD proteins in ascomycete and basidiomycete species, further supporting the view that genes encoding the ancestral apoptotic machinery evolved with early eukaryotes [77-80]. Phylogenetic relationships among the putative PCD associated proteins appear to be complex and many *Aspergillus *proteins show a greater similarity to mammalian than to yeast proteins. In addition, homologs of several mammalian apoptotic proteins including PARP and AMID are found in filamentous fungi, but not in the unicellular yeast such as *S. cerevisiae*, suggesting that the Aspergilli may serve as an alternative model to study mechanisms of cell death.

## Methods

### Sequence similarity

To identify human and mouse proteins implicated in PCD, we searched the Gene Ontology (GO) database [81] and the Apoptosis database [82]. Then, sequence similarity searches were performed using PSI-BLAST and Gapped BLAST against selected fungal genomes downloaded from GenBank. The searches were also performed against an in-house database composed of whole-genome sequences of several fungal species from finished and ongoing sequencing projects. The *N. fischeri *genome sequence has been generated in the course of the genome sequencing projects at TIGR, The Institute for Genomic Research (Rockville, MD). Conserved protein domains were identified using the HMMer package [83].

### Phylogenetic Analysis

Protein sequences were re-aligned using MUSCLE [84] and columns of low conservation removed manually. The alignments were then used to infer bootstrapped neighbor-joining and maximum-likelihood trees. The neighbor-joining trees were constructed using QuickTree [85] and the maximum-likelihood trees were constructed using the PHYLIP package [86], applying the JTT substitution model with a gamma distribution (alpha = 0.5) of rates over four categories of variable sites. In general, the maximum-likelihood and neighbor-joining trees were congruent.

## Authors' contributions

NDF performed the comparative analysis, interpreted the results and prepared the biological aspects of the manuscripts. JHB performed the phylogenetic analysis and allowed use of his Automated Phylogenetic Inference System (APIS). GDR initiated the project and contributed to the comparative analysis. JRW and WCN contributed to the bioinformatics discussion and planning stage of this project and helped drafting the manuscript.

## Supplementary Material

Additional File 1 Putative apoptotic mediators (mammalian protein homologs). Table indicating numbers of fungal homologs of mammalian apoptotic effectors and mediators. Click here for file

Additional File 2 Phylogenetic tree of the AMID family of proteins. Tree reconstruction was performed as described in the Methods section. The numbers indicate percent bootstrap values for internal branches. Click here for file
