# Supplementary material for: Comparative analysis of programmed cell death pathways in filamentous fungi
Source: BMC Genomics. 2005 Dec 8;6:177. doi: 10.1186/1471-2164-6-177 (PMC1325252; doi:10.1186/1471-2164-6-177)
Supplement: Additional File 2 — Phylogenetic tree of the AMID family of proteins. Tree reconstruction was performed as described in the Methods section. The numbers indicate percent bootstrap values for internal branches. [file 1471-2164-6-177-S2.doc]

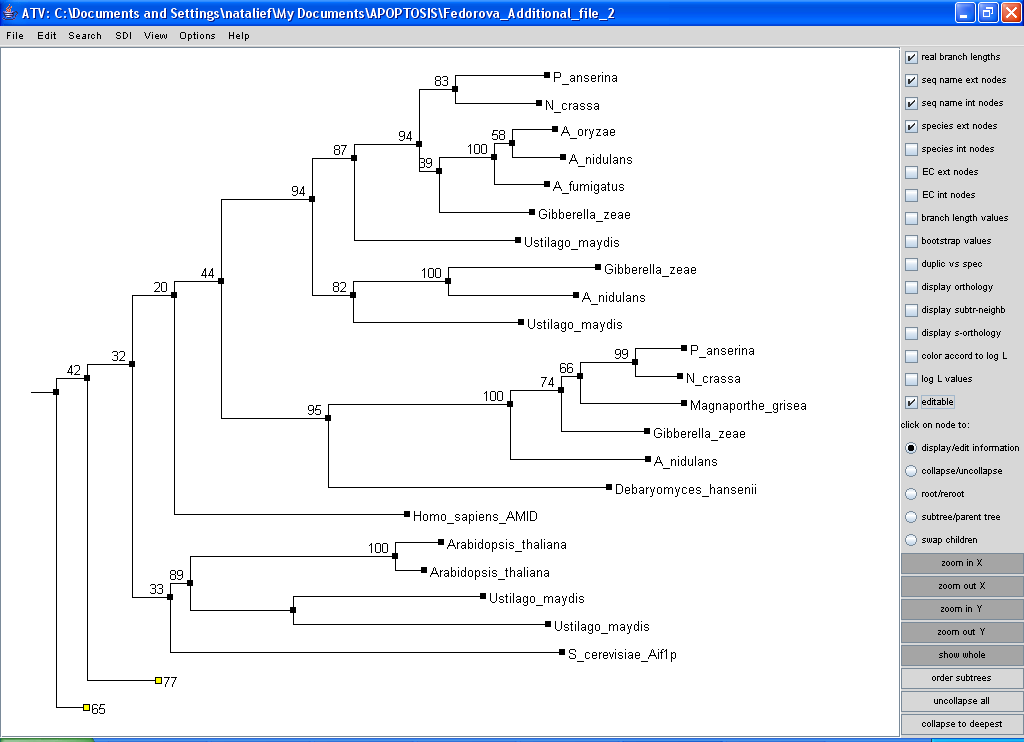


Bacterial and archeal oxidoreductases

Related fungal oxidoreductases

S. cerevisiae Aif1p

Homo sapiens AMID
